# Study on the Association between Ambient Air Pollution and Daily Cardiovascular and Respiratory Mortality in an Urban District of Beijing

**DOI:** 10.3390/ijerph8062109

**Published:** 2011-06-09

**Authors:** Fengying Zhang, Liping Li, Thomas Krafft, Jinmei Lv, Wuyi Wang, Desheng Pei

**Affiliations:** 1 Institute of Geographic Sciences and Natural Resources Research, Chinese Academy of Sciences, 11A Datun Road, Beijing 100101, China; E-Mails: fy-zhang05@hotmail.com (F.Z.); t.krafft@geomed-research.eu (T.K.); lvyuan0403@hotmail.com (J.L.); 2 Graduate University of Chinese Academy of Sciences, Beijing 100049, China; 3 Injury Prevention Research Center, Shantou University Medical College, Shantou 515041, Guangdong Province, China; E-Mails: lipingli65@gmail.com (L.L.); bailiazhu@126.com (D.P.); 4 Department of International Health, Faculty of Health, Medicine and Life Sciences, Maastricht University, 6200 MD Maastricht, The Netherlands

**Keywords:** air pollutants, respiratory disease, cardiovascular disease, mortality, environmental exposure

## Abstract

The association between daily cardiovascular/respiratory mortality and air pollution in an urban district of Beijing was investigated over a 6-year period (January 2003 to December 2008). The purpose of this study was to evaluate the relative importance of the major air pollutants [particulate matter (PM), SO_2_, NO_2_] as predictors of daily cardiovascular/respiratory mortality. The time-series studied comprises years with lower level interventions to control air pollution (2003–2006) and years with high level interventions in preparation for and during the Olympics/Paralympics (2007–2008). Concentrations of PM_10_, SO_2_, and NO_2_, were measured daily during the study period. A generalized additive model was used to evaluate daily numbers of cardiovascular/respiratory deaths in relation to each air pollutant, controlling for time trends and meteorological influences such as temperature and relative humidity. The results show that the daily cardiovascular/respiratory death rates were significantly associated with the concentration air pollutants, especially deaths related to cardiovascular disease. The current day effects of PM_10_ and NO_2_ were higher than that of single lags (distributed lags) and moving average lags for respiratory disease mortality. The largest RR of SO_2_ for respiratory disease mortality was in Lag02. For cardiovascular disease mortality, the largest RR was in Lag01 for PM_10_, and in current day (Lag0) for SO_2_ and NO_2_. NO_2_ was associated with the largest RRs for deaths from both cardiovascular disease and respiratory disease.

## 1. Introduction

It is well established that air pollution is a major threat to human health [1–3]. Numerous time-series studies have indicated a positive association between short-term variation in ambient levels of particulate matter (PM) and daily mortality counts [4–11]. Extensive clinical, epidemiological and toxicological studies have provided evidence of the relationships between exposure to ambient concentrations and human health [12–19]. Even within the limits of the current air quality standards, the negative health effect of air pollutants can still be observed [20–22].

The association between air pollution and deaths from respiratory disease and cardiovascular disease is of general concern to epidemiological researchers [1,23]. In the last decade, many studies have applied time-series methods to search for associations between air pollution and its health effects [10,24–28].

Beijing, as a major metropolis and the capital of China, has a very serious air pollution problem [29–33]. Over the last 30 years, Chinese researchers in the field of environmental health have conducted a series of population studies on the relationship between ambient air pollution and the related health impacts on the people of Beijing. The concentration of PM with aerodynamic diameters less than 10 μm (PM_10_), monitored by the Beijing Environmental Protection Bureau (BJEPB) from 2000 to 2004, indicated that PM was a major problem in Beijing [34]. The risk of cardiovascular mortality was estimated to increase by 11% (95% Confidence Interval [CI]: 5–16%) with each doubling of SO_2_ concentration. The association of total suspended particulates with cardiovascular mortality was positive but not significant [4,35]. Ambient air pollution caused adverse health effects among the exposed population in Beijing during 2000 to 2002 [36].

Most studies have focused on larger areas [6,23,24,35,37], but there has been little research using district-based cardiovascular/respiratory mortality and air pollution data to determine their potential relationship. The district level data provide further evidence on the actual health burden of the urban population by focusing on the inner city and excluding rural Beijing. Most of the earlier research was conducted in the 1990s and in 2000 to 2004 [4,35,36], research conducted during the crucial (policy relevant) period of 2005 to 2008 is still rare.

Despite considerable efforts to improve air quality, air pollution remains the single largest environmental and public health issue affecting Beijing [38–40]. The city’s geographical location intensifies the problem with the surrounding mountain ranges impeding air circulation and dispersion of pollutants [39,40]. The extensive use of coal for providing heating to the rapidly growing population and the unprecedented increase in the number of motor vehicles (approaching 4.03 million vehicles registered in Beijing in February 2010) have outweighed many of the pollution control measures.

This study was undertaken to investigate the relationship between cardiovascular/respiratory mortality and the concentrations of air pollutants in the Chaoyang District of Beijing, over the 6-year period from 2003 through 2008. Chaoyang District was chosen since it is representative of the urban core of Beijing and because of the availability of cardiovascular/respiratory mortality data for the permanent residents. Our study aims for providing further epidemiological and scientific evidence for informed decisions on air pollution control measures.

## 2. Materials and Methods

### 2.1. Study Area and Population

The Chaoyang District, comprising an area of 470.8 km^2^, lies in the east and north east of urban Beijing. The district’s population was 1.522 million people in 2000, and 1.818 million in 2008 [41]. Beijing has a sub-humid warm temperate continental monsoon climate, with annual daily mean temperature of 11.6 °C, minimum mean daily temperature of −4.6 °C in January, and maximum mean daily temperature of 25.9 °C in July.

### 2.2. Data Source

#### Mortality data

The Beijing government requires that a decedent’s family obtain a death certificate from the local public health station (a hospital or a local community clinic) to remove the deceased person from the government-controlled household registration. Also, the decedent’s family must submit the death certificate to the local police station to cancel the decedent’s household registration (*hukou*); thus the decedent’s family obtains two documents (one from the police station and another from the local public health station), which are required before the body can be cremated. The local public health station submits all information from the death certificates to the District Centre for Disease Control and Prevention (CDC) of Beijing. Based on this information the District Centre for CDC of Beijing maintains an electronic death registry.

All mortality data for the calendar years 2003 to 2008 were obtained from death certificates recorded at CDC of Chaoyang District. In the death registry causes are coded by the International Classification of Disease revision 10 (ICD10). For this study, all deaths from cardiovascular disease (CVD) (I00-I99) and respiratory disease (RD) (J00-J98) were identified.

#### Pollutants

Air quality data was provided by the Beijing Municipal Environmental Protection Monitoring Center. Daily ambient air concentrations of PM_10_, SO_2_ and NO_2_ were provided as daily mean values measured from eleven state-controlled monitoring stations in Beijing. According to the technical guidelines of the Chinese government, the location of these monitoring stations must not be in the direct vicinity of traffic intersections or of major industrial polluters and should also have sufficient distance to any other emitting source. Thus the monitoring data reflect the general background urban air pollution level in our study area.

#### Meteorological data

To control for the effects of weather on mortality, meteorological data (daily mean temperature, relative humidity and air/barometric pressure) were obtained from the Beijing Meteorological Office. The weather data were measured at a fixed-site station located in the study district. This station belongs to the Beijing Meteorological Office, the monitoring standard is consistent with international WMO standard, and the data is representative, though small variations in parts of the study area due to the urban micro-climate effect cannot be ruled out. According to the annual temperature of Beijing, we divided the season into the warm season from April to September, and the cool season from October to March (the latter is the heating season, reflected in higher concentrations of some of the air pollutants.).

### 2.3. Data Analysis

The objective of the data analysis was to quantify the association between daily mortality and daily mean air pollutant concentrations, while adjusting for weather and temporal factors in the multivariable modeling. Because the daily number of deaths was small and typically followed a Poisson distribution [28,42–44], the core analysis was a GAM with log link and Poisson error that accounted for fluctuations in daily numbers of deaths. Consistent with other time-series studies [45,46], we used the generalized additive model (GAM) with penalized splines to analyze the daily counts of mortality, air pollution, and covariates (meteorological factors, time trend, and day of the week).

Before conducting the model analyses, there were two steps in the procedure of the model building and model fit: development of the best base model (without a pollutant) and development of the main model (with a pollutant). The latter is achieved by adding the air pollution variables to the final cause-specific best base model, assuming a linear relationship between the logarithmic mortality count and air pollutant concentration.

First, we constructed the basic pattern of mortality excluding the air pollution variables. We incorporated smoothed spline functions of time and weather conditions, which can include non-linear and non-monotonic links between mortality and time/weather conditions, offering a flexible modeling tool [28]. Other covariates, such as day of the week (DOW), were also included in the basic models.

After we established the basic models, we introduced the pollutant variables and analyzed their effects on cardiovascular disease and respiratory mortality. To compare the relative quality of the mortality predictions across these non-nested models, Akaike’s Information Criterion (AIC) was used as a measure of how well the model fitted the data [47]. Smaller AIC values indicate the preferred model. Briefly, we fitted the following log-linear generalized additive models to obtain the estimated pollution log-relative rate β in the study district:

$$\text{log}[E(Y_{t})]=\alpha +\sum_{i=1}^{q}\beta_{i}(X_{i})+\sum_{j=1}^{p}f_{j}(Z_{j},df)+W_{t}(\text{week})$$

Here E(Y_t_) represents the expected number of deaths at day t; β represents the log-relative rate of mortality associated with a unit increase of air pollutants; X_i_ indicates the concentrations of pollutants at day t; W_t_(week) is the dummy variable for day of the week. $\sum_{j=1}^{p}f_{j}(Z_{j},df)$ is the non-parametric spline function of calendar time, temperature and humidity.

Regarding the basic models, we also did some sensitivity analysis following Qian’s method [37]. We initialized the df as 7 df/year for time, 3df for temperature and barometric pressure, 5 df for humidity. We fitted both single-pollutants models and multi-pollutant models (models with a different combination of two or three pollutants per model) to assess the stability of pollutants’ effect.

Further we examined the effect of air pollutants with different lag (L) structures of single day lag (distributed lag; from L0 to L2) and multi-day lag (moving average lag; L01 and L02). Here a lag of 0 day (L0) corresponds to the current-day pollution, and a lag of 1 day refers to the previous-day concentration. In multi-day lag models, L02 corresponds to 3-day moving average of pollutant concentration of the current and previous 2 days [22]. Here, the meteorological factors used in the lag models (distributed lag model, moving average model) were the current day data.

Seasonality was differentiated on the basis of heating/ no-heating periods between the warm season from April to September and October to March as cold season of Beijing with additional pollution from heating sources. Our seasonal analysis followed the method introduced in [42].

All statistical analyses were conducted in R2.9.2 using the MGCV package (R Development Core Team, 2010). The results obtained were expressed as the relative risk (*RR = e*^*βx*Δ*C*^, where Δ*C* is the increased amount of air pollutants, in this study we used 10 μg/m^3^ for comparisons with similiar studies conducted for other places of China) of mortality per 10 μg/m^3^ increase in air pollutant concentrations.

## 3. Results

### 3.1. Descriptive Analysis

The distribution of deaths, meteorological factors, and air pollutants for the study district in Beijing between January 1, 2003 and December 31, 2008 (2,192 days in total) are presented in Table 1.

During the 6-year study period, the mean daily concentrations were 143.07μg/m^3^ for PM_10_, 112.42 μg/m^3^ for SO_2_ and 64.83 μg/m^3^ for NO_2_, respectively. PM_10_ was the major air pollutant in Beijing. The average concentrations of the three air pollutants were below the Grade II national air quality limits (the 24 h mean concentration limit of PM_10_ is 150 μg/m^3^ [48]). However, the maximum daily mean PM_10_ concentration was above the Grade II and even the Grade III national air quality limits; the pollution ranges of PM_10_ were wide, and the upper end was higher than the recommended limits in this study. SO_2_ and NO_2_ also showed some extra high concentrations which exceeded the Grade II national air quality limits (the 24 h mean concentration limit of SO_2_ is 150 μg/m^3^ and of NO_2_ is 80 μg/m^3^ [48]) (Table 2). SO_2_ showed an obvious seasonal variability (Table 1), with peaks in the cold or heating season (October to March). It was also five times higher in the cold than in the warm season, because sulfur rich coal was the major energy source for heating in winter. The average concentration of PM_10_ and NO_2_ showed only small variations between the cold season and the warm season.

Overall, the concentration of air pollutants in Beijing showed an increasing trend from 2003 to 2006, and a decreasing trend in 2007 and 2008 (cf. Table 2). But even with the slight decrease in the later years the air quality in Beijing remained in a rather serious condition. The figures for 2007 and 2008 reflect the air pollution control measures undertaken in preparation for and during the 2008 Olympics/Paralympics [49].

During our study period, the mean daily temperature and humidity were 13.46 °C and 52.68%, respectively. The mean daily temperature ranged from −10.1 °C to 32.1 °C, and the mean daily humidity ranged from 8% to 97%, reflecting the sub-humid warm temperate continental monsoon climate of Beijing.

Table 1 shows the distributions of the daily number of deaths from respiratory disease and cardiovascular disease. From January 1, 2003 to December 31, 2008, a total of 50,032 deaths were recorded, with 22,889 from cardiovascular disease and 4,849 from respiratory disease. On average, there were about 23 deaths per day in our study area, 10 from cardiovascular disease, and two from respiratory disease. In the seasonal-specific distribution, the number of deaths in the cold season was higher than in the warm season.

## 4. Statistical Analysis

The Statistical Package for Social Science, SPSS18.0, was used to analyze the correlation between air pollutants and meteorological factors. Correlation statistics between air pollution parameters and meteorological factors are presented in Table 3.

PM_10_ levels were significantly positively correlated with humidity, negatively correlated with mean air pressure, but had no significant correlation with mean temperature. SO_2_ and NO_2_ levels were significantly positively correlated with mean air pressure and mean humidity but were negatively correlated with mean temperature.

## 5. GAM Analysis

In the one pollutant model, we also took the lag-effect into consideration. Table 4 shows results from the single-lag day for the RR increase in mortality per 10 μg/m^3^ increase in air pollutants.

We found that the current day effects of PM_10_ and NO_2_ were higher than that of single lags (distributed lags) and moving average lags for respiratory disease mortality. The largest RR of SO_2_ for respiratory disease mortality was in Lag02 (three days moving average lag). For cardiovascular disease mortality, the largest RR was in Lag01 for PM_10_, and in current day (Lag0) for SO_2_ and NO_2_.

Among the three air pollutants, NO_2_ was associated with the largest RR for deaths from both cardiovascular disease and respiratory disease. Based on the results from single-pollutants models (Table 4), the largest RRs for respiratory related death were Lag0 for PM_10_ and NO_2_, and the largest RRs of cardiovascular disease mortality were Lag0 for SO_2_ and NO_2_; so we used the current day factors to run the multiple-pollutants models for respiratory disease mortality and cardiovascular disease mortality. The results are shown in Table 5.

We observed a significant relationship between deaths from cardiovascular/respiratory diseases and the three air pollutants in both single pollutant models and multiple pollutant models. For deaths from respiratory disease, the effects of PM_10_ decreased after adding SO_2_ and NO_2_ (Table 5). The effects of SO_2_ on respiratory disease mortality did markedly change after adding PM_10_ into the model. The effects of NO_2_ increased markedly after adding SO_2_ or PM_10_. In the three air pollutants model, both the effects of PM_10_ and SO_2_ decreased markedly, but the effects of NO_2_ increased.

The effects of PM_10_ on cardiovascular disease mortality increased when NO2 was added to the two pollutants model, but did not markedly change after adding SO_2_ into the model. The effects of SO_2_ on cardiovascular disease mortality decreased after adding PM_10_ and NO_2_ into the models. The effects of NO_2_ on cardiovascular disease mortality showed the same trend as for SO_2_. In the three pollutants model for cardiovascular disease mortality, the effects of PM_10_ increased; both SO_2_ and NO_2_ showed a decreasing trend.

PM_10_ concentrations had a higher effect on deaths from cardiovascular disease than on respiratory disease ones. SO_2_ had a similar effect both on deaths from cardiovascular disease and deaths from respiratory disease. NO_2_ had greater effects on respiratory disease mortality than on cardiovascular disease mortality.

The seasonal analysis results shown higher mortality risks related to PM_10_ and SO_2_ during cold times for both the respiratory disease and the cardiovascular disease than that during warm times. For SO_2_, the RRs with 10 μg/m^3^ increasing of concentration were higher during warm season than that in cold season (Table 6).

## 6. Discussion

Our study combined epidemiological and environmental health science research methods to study associations between major air pollutants and deaths from cardiovascular disease and respiratory disease over a period of six years. The findings have implications for environmental and social policies in the study district and beyond. The results showed that deaths from cardiovascular disease and respiratory disease were increased on days of greater air pollution. RR estimates for PM_10_ in Lag0,1,2 and Lag01,02 were significant associated with both the cardiovascular disease mortality and the respiratory disease mortality. Particulate matters have been indentified to have effect on respiratory mortality and respiratory mortality, and several potential mechanisms have been indicated [14].

The health effects showed different lag times for various pollutants in our study (Table 4). This is in accordance with other air pollution mortality studies in the Asian region [50]. In the single pollutant model, the independent health effects of PM_10_ and NO_2_ were higher than SO_2_ for both respiratory disease mortality and cardiovascular disease mortality. A study conducted by Xu *et al*. found that SO_2_ was associated with daily mortality in Beijing [4]. With the rapid increase in the number of motor vehicles in recent years, outdoor air pollution in Beijing has gradually changed from the conventional coal combustion type to the mixed coal combustion/motor vehicle emission type. We also found that PM_10_ had a relationship with cardiovascular disease mortality. This was in accordance with other studies [4–7].

Significant effects were more likely to be seen during October through March than during the warm season for both disease groups. Wind speed is inversely related to air pollution levels, and rain can modify the composition of air pollutants, while sun irradiation induces photochemical modifications of several pollutants. A recent study conducted in Shanghai found that several pollutants had a more significant impact on daily hospital admissions in the cold season than in the warm season [22]. A study conducted in Hong Kong also showed similar results [50].

A 1990s study conducted by Yang *et al*., found significant associations between cardiovascular mortality and the three main air pollutants in the single-pollutant model [51]. An increase of 10 μg/m^3^ for PM_10_, SO_2_, NO_2_, corresponded to 1.004, 1.004, 1.013 RR in cardiovascular mortality. Compared with the results of studies conducted by Xu *et al*. in the 1990s and Yang *et al.* in 2003 (Table 7), the associations between air pollutants and cardiovascular mortality showed a relative curvature, implying a reduction of the negative effects on health caused by ambient air pollution in the urban areas of Beijing in recent years [36]. Since 1998 the local government of Beijing has invested considerable money and implemented a host of measures and policies aimed at improving the air quality in Beijing city and its environs. Over time, the air quality has improved gradually after introduction of the following measures: coal desulfurization, changes in the public transport system and road traffic control, and change of energy use patterns. The annual levels of ambient SO_2_, NO_2_ and PM_10_ were 69, 49 and 122 μg/m^3^, respectively in 2008; exhibiting a reduction of 43.8%, 30.9% and 26.1%, respectively, from the 2001 levels [36]. Higher living standards, better hygienic conditions and better medical care can reduce the number of deaths, and the control of air pollution is also likely to result in health benefits [22]. However, also as a result of the rapid increase in road traffic the air quality remains to be critical and still needs further improvement.

Compared with previous studies conducted in Europe, USA and elsewhere (Table 7), our epidemiological study, with some exceptions, reports lower coefficients in the exposure-response functions for air pollution and health effects. The health effect of PM_10_ on cardiovascular disease and respiratory disease mortalities was similar to that reported in the USA [52–54], The Netherlands, and Vienna, but lower than in Hong Kong [50]. This may be related to the chemical composition of PM_10_, the age of the exposed population, the citizens’ sensitivity to air pollution, *etc* [4]. Long-term high levels of air pollution can increase the adaptability of the population. Inorganic matter is the main component of PM_10_ in Beijing, and has low toxicity [4]. PM_10_ in developed countries and regions is mainly from vehicle exhausts, which has high toxicity on human health [55].

The health effect of NO_2_ on these two disease groups in our study was also higher than in other studies conducted in America [53], The Netherlands [54], and Vienna [52], but lower than in Hong Kong [50]. NO_2_ is the main product of automobile exhaust fumes. The increased cardiovascular mortality risks observed in the Chinese population are similar in magnitude, per quantity of pollution, to the risk found in other parts of the World, but air pollution in China is at much higher levels in general, and the effect of pollutants on cardiovascular risk is greater than in North America or Europe. The higher toxicity of NO_2_ may be the results of increasing car ownership in urban areas (by the end of 2008, car ownership in Beijing had reached 2.483 million), causing the proportion of NO_2_ in air pollutant levels to be increased [56].

In our study, it was found that urban air pollution can increase cardiovascular mortality and respiratory mortality among citizens in Chaoyang District of Beijing. These findings also confirm the effects of air pollutants (particulate matters, nitrogen dioxide, sulfur dioxide) described in other studies [50,51].

Our study has some limitations. For want of more detailed data, we averaged the monitoring results of 11 official monitoring stations as the proxy for the population exposure level to air pollution. This simple averaging method may raise a number of issues, because the pollutant measurements can differ between locations and the ambient monitoring results may differ from the personal exposure level to air pollutants. The error in estimating personal exposure based on fixed site monitoring stations would tend to reduce the probability of detecting an effect and introduce bias into the air pollution exposure-mortality relationship. Further studies on personal exposure should be conducted.

It was difficult to isolate the effects of one pollutant in this specific study, because of the high correlations between the three pollutants. Further and more detailed studies are needed to clarify the findings in this one.

PM_2.5_ is a better indicator of air pollution and has a higher health risk than PM_10_ [29]. Though the PM_2.5_/PM_10_ ratio (ratio = concentration of PM_2.5_/concentration of PM_10_) has been established in Beijing. The reported ratios varied spatially and temporally, ranging from 0.25 to 0.8 [29]. For daily concentration of PM_2.5_ for our time period was not available, this was a limitation when assessing the health effects of particulate matters. In summary, air pollutant levels remaining within the limits of current air quality standards in residential areas in Beijing, an apparent health effect of air pollutants can still be observed.

## Figures and Tables

**Table 1.** Daily pollutant concentrations, meteorological factors and numbers of deaths.

		***Mean***	***Warm***	***Cold***	***SD***	***Percentage***				
						**Min**	**25**	**Median**	**75**	**Max**
Air pollutants concentration 24 h mean (μg/m^3^)	PM_10_	143.1	138.1	148.1	87.2	9.0	82.0	128.0	180.0	600.0
	SO_2_	112.4	22.9	202.3	316.9	5.0	17.0	30.0	64.0	1643.0
	NO_2_	64.8	58.2	71.5	24.2	14.4	49.6	62.4	78.4	214.4

Meteorological measures 24 h mean	Temperature (°C)	13.5	22.6	4.3	10.9	−10.1	3.2	14.7	23.5	32.1
	Humidity (%)	52.7	58.5	46.8	20.2	8.0	36.0	54.0	69.0	97.0
	Air pressure (hPa)	1012.6	1004.8	1020.5	101.8	987.8	1004.5	1012.4	1020.8	1043.0

Daily deaths, mean	Total	22.8	21.1	24.6	7.2	6.0	18.0	22.0	27.0	54.0
	Cardiovascular	10.4	9.4	11.5	4.0	1.0	8.0	10.0	13.0	27.0
	Respiratory	2.2	1.9	2.5	1.8	0.0	1.0	2.0	3.0	14.0

**Table 2.** Number of days/per annum with air pollutants exceeding the standard limits and annual average concentration of the pollutants.

	***PM****_10_****(μg/m****^3^****)***			***SO****_2_****(μg/m****^3^****)***			***NO****_2_****(μg/m****^3^****)***		

***Year***	***Grade II (≥150)***	***Grade III (≥250)***	***Annual average concentration***	***Grade II (≥150)***	***Grade III (≥250)***	***Annual average concentration***	***Grade II (≥80)***	***Grade III (≥120)***	***Annual average concentration***
2003	133	27	136.05	26	26	129.12	117	5	70.24
2004	134	42	141.61	8	8	73.55	93	14	69.47
2005	135	43	148.97	21	21	128.28	84	12	66.59
2006	127	51	160.66	28	28	156.47	93	13	66.59
2007	127	47	149.11	19	19	117.58	85	5	66.38
2008	94	28	122.06	9	9	69.75	44	2	49.77
Total	750	238		111	111		516	51	

Note: Air Quality Standards: Grade I for areas such as nature reserves and other areas that need special protection. Grade II is the standard for mainly residential area, commercial areas and mixed use urban areas as well as the rural areas. Grade III standard applies to specific industrial zones [48].

**Table 3.** Pearson coefficients of daily deaths, air pollutants and meteorological factors.

	***PM****_10_*	***SO****_2_*	***NO****_2_*	***Mean air pressure***	***Mean temperature***	***Mean humidtity***
PM_10_	1.000	0.308	0.615	−0.105	0.003	0.168
SO_2_	0.308	1.000	0.426	0.248	−0.360	0.056
NO_2_	0.615	0.426	1.000	0.128	−0.186	0.207

*n* = 2,192.

**Table 4.** Distribution of RR across lags of different pollutants for respiratory disease and cardiovascular disease death.

***Respiratory***	***PM****_10_*		***SO****_2_*		***NO****_2_*	

	**RR**	**95% CI**	**RR**	**95% CI**	**RR**	**95% CI**
Lag0	1.00101	1.00057–1.00145	1.00029	1.00018–1.00039	1.00947	1.00759–1.01135
Lag1	0.99967	0.99908–1.00027	1.00002	0.99992–1.00012	0.99989	0.99828–1.00149
Lag2	0.99883	0.99746–1.00020	1.00049	1.00039–1.00059	1.00203	1.00056–1.00350
Lag01	1.00038	0.99988–1.00088	1.00024	1.00011–1.00037	1.00619	1.00402–1.00836
Lag02	0.99946	0.99701–1.00011	1.00063	1.00047–1.00078	1.00656	1.00421–1.00891

***Cardiovascular***	***PM****_10_*		***SO****_2_*		***NO****_2_*	

	**RR**	**95% CI**	**RR**	**95% CI**	**RR**	**95% CI**

Lag0	1.00164	1.00144–1.00184	1.00022	0.99917–1.00127	1.00271	1.00086–1.00457
Lag1	1.00098	1.00079–1.00116	1.00001	0.99896–1.00106	0.99455	0.98782–1.00129
Lag2	0.99926	0.99809–1.00043	0.99982	0.99877–1.00087	0.99679	0.99312–1.00047
Lag01	1.00187	1.00164–1.00211	1.00019	1.00012–1.00025	0.99698	0.99200–1.00197
Lag02	1.00096	1.00070–1.00121	1.00006	0.99999–1.00014	0.99508	0.990010–1.00015

**Table 5.** RR for a 10 μg/m^3^ increase in pollutant levels for respiratory disease mortality and cardiovascular disease mortality.

***Model***	***Pollutant***	***Respiratory disease***		***Cardiovascular disease***	

		**RR**	**95% CI**	**RR**	**95% CI**
Single pollutant	PM_10_	1.00101	1.00057–1.00145	1.00164	1.00144–1.00184
	SO_2_	1.00029	1.00018–1.00039	1.00022	0.99917–1.00127
	NO_2_	1.00947	1.00759–1.01135	1.00271	1.00086–1.00457

Two-pollutant	PM_10_	0.99974	0.99922–1.00027	1.00181	1.00157–1.00205
	NO_2_	1.01005	1.00782–1.01228	0.99866	0.99765–0.99967

	PM_10_	1.00065	1.00018–1.00113	1.00152	1.0013–1.00173
	SO_2_	1.00023	1.00012–1.00034	1.00009	1.00004–1.00015

	NO_2_	1.00882	1.00675–1.01089	1.00155	1.00062–1.00247
	SO_2_	1.00008	0.99997–1.00020	1.00018	1.00013–1.00024

Three-pollutant	PM_10_	0.99966	0.99913–1.0002	1.00173	1.00148–1.00197
	NO_2_	1.00949	1.00716–1.01182	0.99807	0.99603–1.00012
	SO_2_	1.00010	0.99998–1.00021	1.00012	1.00007–1.00018

Note: here the RRs of single pollutant were the results of current day analysis.

**Table 6.** RR for a 10 μg/m^3^ increase in pollutant levels in seasonal specified analysis.

		**PM_10_**		**SO_2_**		**NO_2_**	
*Respiratory disease*	warm	0.99903	0.99730–1.00076	1.01648	1.01140–1.02157	0.99510	0.98930–1.00090
	cold	1.00252	1.00217–1.00307	1.00079	1.00049–1.00109	1.01692	1.01638–1.01746

*Cardiovascular disease*	warm	1.00077	1.00045–1.00108	1.01621	1.01406–1.01836	1.00042	0.99883–1.00201
	cold	1.00271	1.00254–1.00318	1.00030	1.00021–1.00039	1.00911	1.00897–1.00925

**Table 7.** Comparison of RR for a 10 μg/m^3^ increase in pollutant concentration worldwide.

***Area***	***Year***	***Disease***	***PM****_10_*		***SO****_2_*		***NO****_2_*	

			**RR**	**95% CI**	**RR**	**95% CI**	**RR**	**95%CI**
Beijing (current study)	2003–2008	CVD	1.00164	1.00144–1.00184	1.00022	0.99917–1.00127	1.00271	1.00086–1.00457
		RD	1.00101	1.00057–1.00145	1.00029	1.00018–1.00039	1.00947	1.00759–1.01135
Beijing [51]	2003	CVD	1.004	1.002–1.006	1.004	1.001–1.008	1.013	1.002–1.024
American [53]	1987–2000	CVD&RD	1.0024	1.0013–1.0036				
Netherlands [54]	1992–2006	CVD	1.002	1.001–1.004			1.008	1.005–1.011
		RD	1.004	1.002–1.007			1.008	1.003–1.012
Hong Kong [50]	1996–2002	CVD	1.0058	1.0014–1.0103	1.0103	1.0021–1.0185	1.0117	1.0061–1,0173
		RD	1.0089	1.0036–1.0142	1.0106	1.0006–1.0206	1.0092	1.0025–1.016
Vienna [52]	2000–2004	CVD	1.002	1.0009–1.0031			1.0046	1.0029–1.0063
		RD	1.0035	1.0001–1.0069			1.0067	1.0027–1.0108
